# Recent Development of Polymer Nanofibers in the Field of Optical Sensing

**DOI:** 10.3390/polym15173616

**Published:** 2023-08-31

**Authors:** Jinze Li, Xin Liu, Jiawei Xi, Li Deng, Yanxin Yang, Xiang Li, Hao Sun

**Affiliations:** 1 School of Optoelectronic Engineering, Xidian University, Xi’an 710071, China; lijinze@xidian.edu.cn (J.L.); jiaweixi@stu.xidian.edu.cn (J.X.); ldeng@stu.xidian.edu.cn (L.D.); yangyanxin@stu.xidian.edu.cn (Y.Y.); xiangli@xidian.edu.cn (X.L.); 2 School of Physics, Xidian University, Xi’an 710071, China; liuxin0327@stu.xidian.edu.cn

**Keywords:** polymer nanofiber, optical sensor, biomedicine, environment monitoring, food safety

## Abstract

In recent years, owing to the continuous development of polymer nanofiber manufacturing technology, various nanofibers with different structural characteristics have emerged, allowing their application in the field of sensing to continually expand. Integrating polymer nanofibers with optical sensors takes advantage of the high sensitivity, fast response, and strong immunity to electromagnetic interference of optical sensors, enabling widespread use in biomedical science, environmental monitoring, food safety, and other fields. This paper summarizes the research progress of polymer nanofibers in optical sensors, classifies and analyzes polymer nanofiber optical sensors according to different functions (fluorescence, Raman, polarization, surface plasmon resonance, and photoelectrochemistry), and introduces the principles, structures, and properties of each type of sensor and application examples in different fields. This paper also looks forward to the future development directions and challenges of polymer nanofiber optical sensors, and provides a reference for in-depth research of sensors and industrial applications of polymer nanofibers.

## 1. Introduction

Optical sensors use light signals for detection and measurement. They have the advantages of high sensitivity, fast response speed, and strong immunity to electromagnetic interference, and are widely used in biomedical, environmental monitoring, food safety, energy conversion and other fields [1,2,3,4,5]. With the development of optical sensing, great demands have been made for their sensing materials and device performance. Polymer nanofibers, as a green nanomaterial derived from nature, have attracted great interest from researchers due to their wide availability, diverse preparation methods, biodegradability, safety and nontoxicity, high specific surface area, high strength, low density, and good thermal stability [6,7,8,9,10,11,12]. This article reviews the research progress of polymer nanofibers in optical sensors, and classifies and analyzes nanofiber optical sensors according to different functions (fluorescence [13], Raman [14], polarization [15], surface plasmon resonance [16], photoelectrochemistry [17], and fluorescence resonance energy transfer [18]). In addition, we introduce the principles, structures, and properties of each type of sensor with examples of applications in different fields.

**Figure 1:**
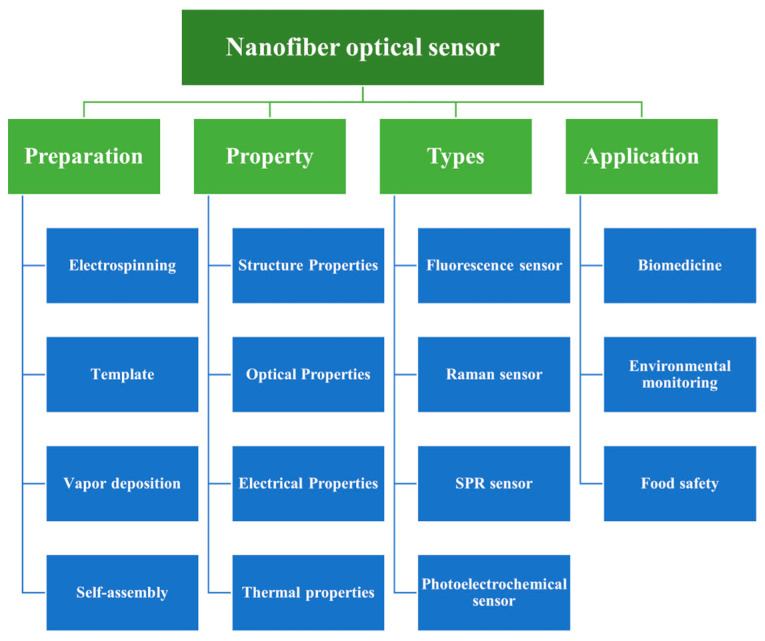
Main content of nanofiber optical sensor research.

Polymer nanofibers have excellent film-forming properties and abundant surface-active functional groups, which make them suitable for further processing into two-dimensional (membrane materials) and three-dimensional materials (such as hydrogels and aerogels) [19,20,21,22]. Polymer nanofibers with different dimensions have different characteristics, which provide great flexibility for the design of nanofiber optical sensors to meet the design requirements of different optical sensors. The unique properties of nanofibers make them suitable for different types of optical applications, including physical, biological, and chemical, where they perform different roles, for example, as dispersants, carriers, and templates [23,24,25,26,27]. As shown in Figure 1, this review examines the research progress made in polymer nanofibers in optical sensors, compares and summarizes the advantages and disadvantages of nanofiber sensors and their applications, anticipates the future development directions and challenges for nanofiber optical sensors, and provides a reference for in-depth research on nanofiber optical sensors.

## 2. Preparation Methods and Properties of Nanofibers

### 2.1. Preparation Methods of Nanofibers

Polymer nanofibers can be prepared using various methods such as electrospinning, templating, vapor deposition, and self-assembly.

#### 2.1.1. Electrospinning

**Figure 2:**
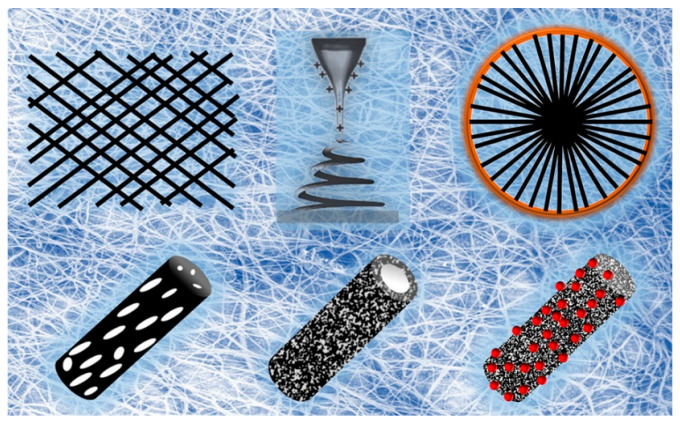
Preparation of nanofibers by electrospinning. Reproduced with permission from reference [34]. Copyright 2017, American Chemical Society.

Electrospinning is a simple and effective method for preparing nanofibers, as shown in Figure 2. It involves injecting a polymer solution into an electrospinning device and then spraying the solution under high pressure to form nanofibers. Electrospinning is one of the simplest top-down methods for the preparation of polymer nanofibers. Electrospinning can produce nanofibers with diameters ranging from several nanometers to hundreds of micrometers, which have good continuity and uniformity [28,29,30,31,32,33]. Electrospinning is the most widely used method for preparing nanofibers, owing to its simple instrumentation, low cost, and scalability.

#### 2.1.2. Template Method

**Figure 3:**
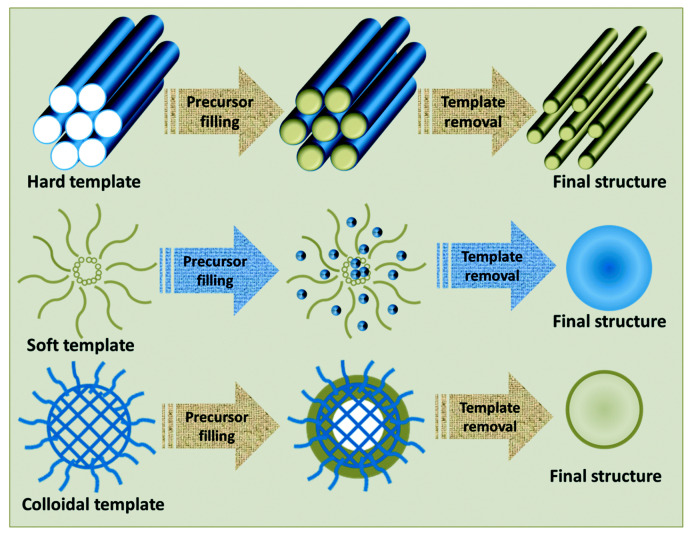
Preparation of nanofibers by the template method. Reproduced with permission from reference [39]. Copyright 2020, Royal Society of Chemistry.

As shown in Figure 3, the template method is a bottom-up method that involves injecting a polymer solution into the pores of a template, then drying it at high temperature, and finally removing the template to obtain the nanofibers. The template method can produce nanofibers with diameters ranging from tens to hundreds of nanometers, which have good morphology and structure [35,36,37,38]. However, the nanofibers prepared by the template method are not scalable.

#### 2.1.3. Vapor Deposition Method

**Figure 4:**
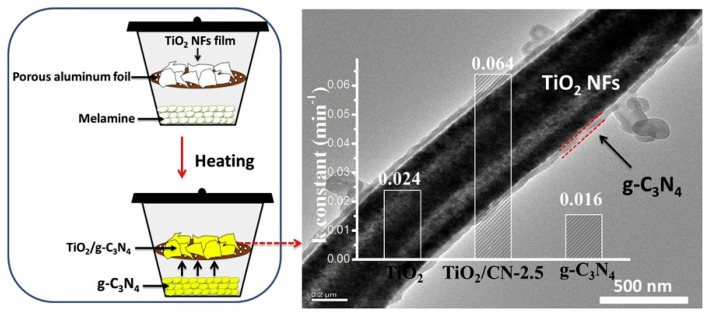
Preparation of nanofibers by vapor deposition. Reproduced with permission from reference [43]. Copyright 2020, Elsevier.

As shown in Figure 4, the vapor deposition method involves evaporating metal or semiconductor materials into a high-temperature reaction chamber to form gas-phase particles, and then depositing nanowire fibers on the substrate. The vapor deposition method can produce nanofibers with diameters ranging from tens to hundreds of nanometers, which have good crystal structure and morphology [40,41,42]. The vapor deposition method is a top-down method that involve the deposition of vaporized materials onto a substrate. However, the vapor deposition method consumes a lot of energy, and the nanofibers are uneven and easy to agglomerate.

#### 2.1.4. Self-Assembly Method

**Figure 5:**
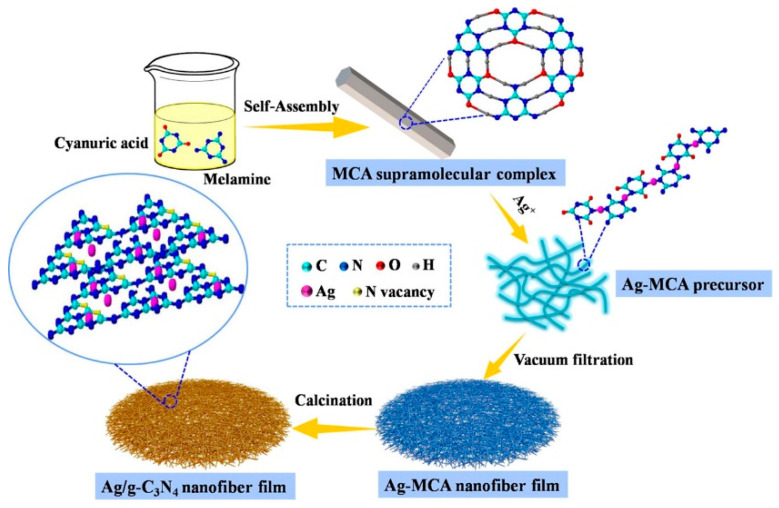
Preparation of nanofibers by the self-assembly method. Reproduced with permission from reference [47]. Copyright 2021, American Chemical Society.

The self-assembly method is a bottom-up nanomaterial fabrication method in which molecules organize and arrange themselves into patterns or structures through noncovalent forces, such as hydrogen bonding, hydrophobic forces, and electrostatic interactions. As shown in Figure 5, the self-assembly method can be used to prepare nanofibers of different materials, such as peptides, block copolymers, liquid crystal polymers, etc. The self-assembly method can produce nanofibers with diameters ranging from a few nanometers to 100 nanometers. The self-assembly method has some advantages over other methods for preparing nanofibers, such as low cost, simple operation, mild conditions, and high versatility [44,45,46]. However, the dimensions of the nanofibers produced by the self-assembly method are hard to control.

Among these methods for polymer nanofiber preparation, electrospinning has the lowest cost and the highest efficiency. By adjusting the relevant parameters in the electrospinning process, various forms of nanofiber can be prepared and applied to optical sensing. Therefore, in the future, efficiently preparing various polymer nanofibers that can be easily integrated into optical sensing platforms, and can improve their performance, will be a primary research focus.

### 2.2. Properties of Nanofibers

#### 2.2.1. Structure Properties

**Figure 6:**
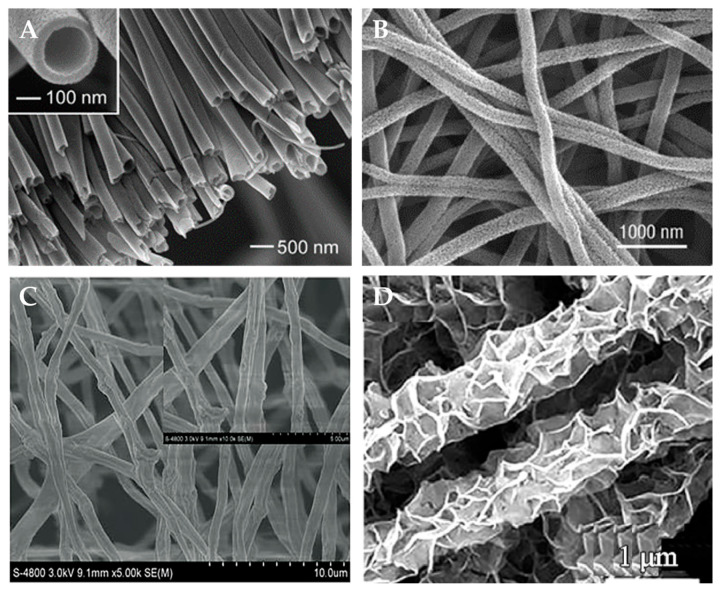
Polymer nanofibers with different structures and morphologies. (**A**) Hollow nanofiber. Reproduced with permission from reference [48]. Copyright 2004, American Chemical Society. (**B**) Rough nanofiber. Reproduced with permission from reference [51]. Copyright 2019, Wiley. (**C**) Wrinkled nanofiber. Reproduced with permission from reference [52]. Copyright 2022, Frontiers. (**D**) Barbed nanofiber. Reproduced with permission from reference [53]. Copyright 2021, Elsevier.

Polymer nanofibers have high specific surface area, low density, and high porosity and pore connectivity, which endow them with good adsorption performance, filtration performance, and moisturizing performance. As shown in Figure 6, nanofibers have various morphologies, such as hollow, rough, wrinkled, and barbed. Polymer nanofibers also possess excellent mechanical properties such as high tensile strength, elastic modulus, and toughness, which confer them with good heat, wear, and corrosion resistances. Moreover, polymer nanofibers have abundant surface functional groups, such as hydroxyl groups and carboxyl groups, which impart to them both good chemical stability and chemical modifiability, allowing them to form composite materials, or to be functionalized with other materials [48,49,50,51].

#### 2.2.2. Optical Properties

**Figure 7:**
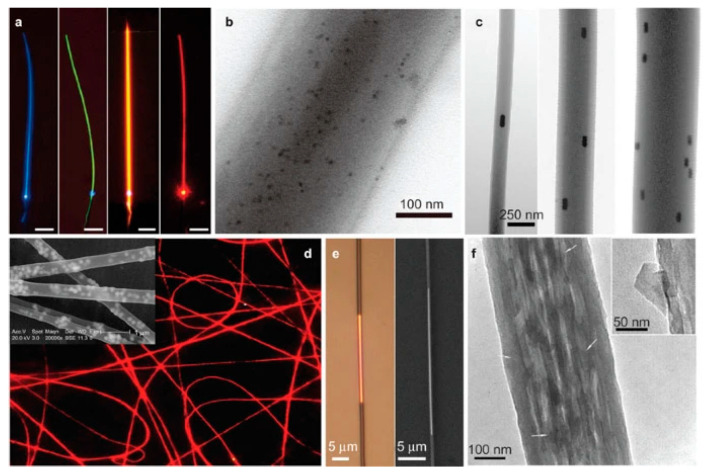
Optical properties of polymer nanofibers. Reproduced with permission from reference [58]. Copyright 2013, Springer. (**a**) Typical light-emitting polymer nanofibers excited by 355-nm light. (**b**) A 280 nm diameter PS nanofiber doped with CdSe quantum dots. (**c**) Three PAM nanofibers doped with aligned GNRs. (**d**) Fluorescent microscopy image of PVA nanofibers doped with Fe2O3 nanoparticles and a europium complex. (**e**) Optical microscope and SEM images of a PMMA nanofiber doped with a silver nanowire. (**f**) High-magnification TEM image of a graphene-doped PVA nanofiber.

Owing to their special structural properties, polymer nanofibers can exhibit quantum confinement effects. Furthermore, the structures of polymer nanofibers endow them with special optical properties such as optical transmittance, optical interference, scattering, and liquid crystal chirality. Optical transmittance: the nanofibers have a high optical transmittance and can be used as a transparent substrate or film material, such as for solar cells, displays, or sensors. Optical interference: the nanofibers have a high birefringence and can produce colorful interference effects, which can be used in color displays or reflective coating materials. Scattering: the nanofibers have a high scattering intensity, which can enhance the light capture efficiency of optoelectronic devices, and can also be used in scattering-type displays or reflective coating materials. Liquid crystal chirality: the nanofibers are a natural chiral material, which can self-organize into nematic and cholesteric liquid crystals, and exhibit structural color and polarization-selective reflection, which can be used as chiral photonic crystals or optical coding materials. Figure 7 shows the optical properties of different polymer nanofibers after doping with GNR, quantum dot materials, and nanomaterials. These special properties can be utilized in the chiral photonics, flexible electronic device, energy storage device, and information encryption fields [54,55,56,57].

#### 2.2.3. Electrical Properties

**Figure 8:**
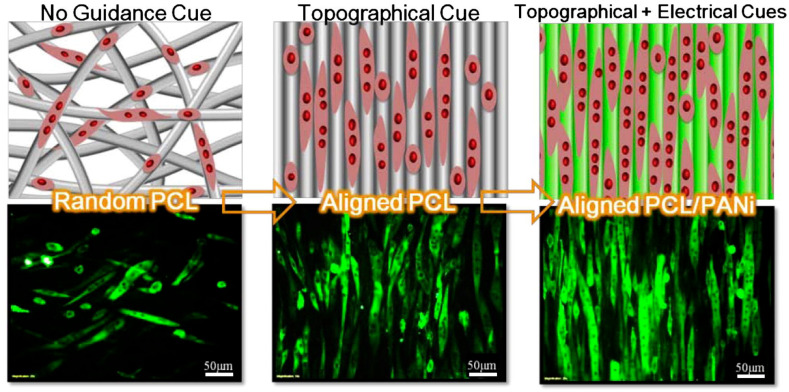
Electrical properties of nanofibers. Reproduced with permission from reference [63]. Copyright 2013, Elsevier.

Owing to the unique structural properties of polymer nanofibers, they exhibit special electrical properties, such as conductivity, electrochromic, piezoelectric effect, etc. Conductivity: nanofiber conductivity can be improved by doping with conductive materials or coating with conductive layers, allowing the nanofibers to be used as sensors, electrodes, or supercapacitors. Electrochromic behavior: the nanofibers can produce different colors under different voltages by controlling their structure or composition, which can be used in displays and smart windows. Piezoelectric effect: the nanofibers can generate electric charge by being subjected to mechanical pressure, or produce deformation by applying voltage, which can be used in generators, actuators, and sensors. Figure 8 shows the effects of the electrical properties of nanofibers with different orientations on cells. The different electrical properties of nanofibers mean they are widely used in energy storage devices, wearable or bendable electronic devices, information encryption, optical coding, optical data storage, environmental governance, and as water treatment filtration barriers [59,60,61,62].

#### 2.2.4. Thermal Properties

**Figure 9:**
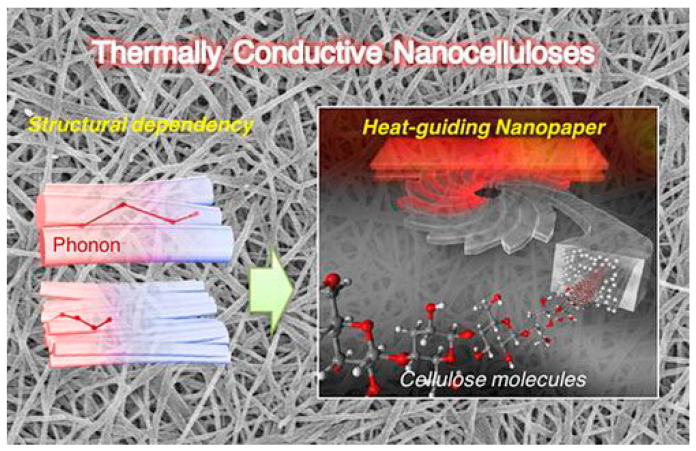
Thermal properties of nanofibers. Reproduced with permission from reference [68]. Copyright 2017, Taylor and Francis.

Due to the various shapes of polymer nanofibers and the differences in their constituent materials, they can exhibit special thermal properties, such as thermal conductivity, thermal expansion, and thermal stability. Thermal conductivity: nanofibers have low thermal conductivity and can be used as insulation or thermal insulation materials, such as aerogels and foams. Thermal expansion: nanofibers have a low thermal expansion coefficient and can be used as high-temperature stable structural materials, such as composites and ceramics. Thermal stability: nanofibers have high thermal stability and can withstand the influence of high temperatures or flames, are not easily deformed or degraded, and can be used as fire-proof or high-temperature resistant materials. Figure 9 shows the different thermal properties of nanofibers that make them widely applicable in the chemical, pharmaceutical, energy, and aerospace industries [64,65,66,67].

## 3. Nanofiber Optical Sensors

Polymer nanofiber optical sensors are a type of optical sensor that utilize nanoscale fiber materials as sensitive elements or signal conversion media. Polymer nanofiber optical sensors can measure the interaction of light with matter, for example, absorption, scattering, fluorescence, and Raman, which allows monitoring of the physical and chemical properties of matter, including the temperature, pressure, humidity, pH, and presence of biomolecules. Polymer nanofiber optical sensors can be classified into fluorescence, Raman, surface plasmon resonance, and photoelectrochemical sensors, according to their different functions.

### 3.1. Fluorescence Sensor

Fluorescence sensors are sensors that utilize fluorescent substances to emit fluorescence of a specific wavelength when exposed to excitation light, and the fluorescence intensity or wavelength correlates with the measured substance or signal. Fluorescence sensors have the advantages of high sensitivity, good selectivity, and high signal-to-noise ratio, as shown in Table 1, and can be used for detection in biology [69], chemistry [70], environmental monitoring [14], and other fields [13,71,72].

**Figure 10:**
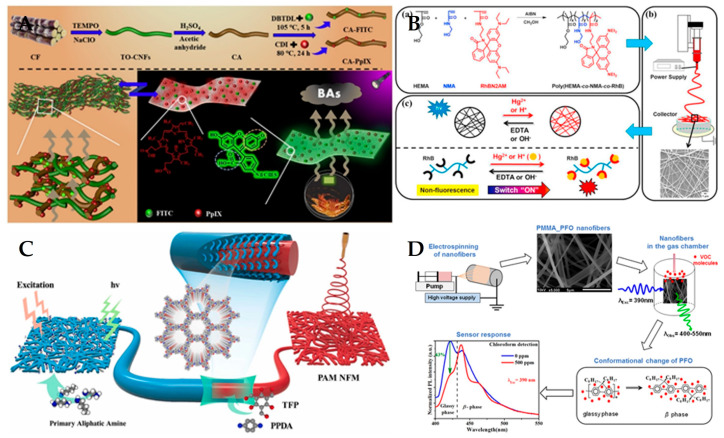
Different types of polymer nanofiber fluorescent sensors. (**A**) A nanofiber fluorescent sensor for detecting the freshness of shrimp and pork. Reproduced with permission from reference [84]. Copyright 2021, Elsevier. (**B**) A nanofiber fluorescent sensor pH and Hg^2+^. Reproduced with permission from reference [86]. Copyright 2017, Elsevier. (**C**) A fluorescent sensor for primary aliphatic amine gas. Reproduced with permission from reference [78]. Copyright 2022, Elsevier. (**D**) A nanofiber fluorescent sensor for gaseous volatile organic compounds. Reproduced with permission from reference [87]. Copyright 2017, Elsevier.

In recent years, many studies have reported the development and application of polymer nanofiber fluorescent sensors for various applications, such as for detecting the freshness of shrimp and pork, pH and Hg^2+^, aliphatic amine gas, and volatile organic compounds, as shown in Figure 10. (1) For pH detection, Yao et al. [73] prepared a color-tunable luminescent nanofiber for pH and glucose sensing based on CdTe quantum dots-loaded bacterial cellulose nanofibers. Xu et al. [74] synthesized a 1,8-naphthimide-based polymer/poly (vinyl alcohol) electrospinning nanofiber membrane for pH fluorescence sensing. (2) For gas detection, Zhou et al. [75] adjusted the inter-nanofiber spacing in the bundled nanofibers for volatile organic compound detection based on polyvinylpyrrolidone and rhodamine B. Luo et al. [76] developed a nanofiber fluorescent hydrogel for chromium (VI) detection based on chitosan, titanate, and cellulose nanofibers modified with carbon dots. Wang et al. [77] developed an instant-response nanofiber fluorescent sensor for phosgene detection based on polyacrylonitrile and 2-(2-hydroxyphenyl) benzoxazole derivatives. Zhao et al. [78] fabricated a covalent organic framework modified polyacrylamide electrospinning nanofiber membrane as a “turn-on” fluorescent sensor for primary aliphatic amine gas. Hu et al. [72] designed a nanofiber fluorescent sensor for phosgene detection based on small organic dyes and polyacrylonitrile. (3) For biomolecules detection, Yang et al. [79] constructed a networked fluorescence aptasensing platform for biomarkers detection based on quantum dots and electrospinning nanofibers. Ahmadian-Fard-Fini et al. [80] produced a nanofiber fluorescent sensor for mercury (II) and lead (II) ions detection based on cellulose acetate nanofibers and carbon dots. Chen et al. [81] proposed an electrospinning nanofiber platform for nerve gas mimic detection based on an excited-state intramolecular proton transfer fluorescent probe. (4) For explosives detection, Liao et al. [82] fabricated a nanofiber fluorescent sensor for nitro-based explosives detection based on oligotriphenylene and polystyrene. George et al. [83] presented a reversible fluorescent probe for nitro and peroxide organic explosives detection based on electrospinning barium tungstate nanofibers. (5) For biogenic amines detection, Quan et al. [84] prepared a nanofiber ratiometric fluorescent sensor for biogenic amines detection based on cellulose nanofibers and fluorescein isothiocyanate. Xu et al. [85] synthesized N-doped graphene quantum dots from bulk N-doped carbon nanofiber film for iron (III) and ascorbic acid detection. According to these studies, the advantages of nanofiber fluorescent sensors are: first, they have high sensitivity and low detection limits due to the strong evanescent field and tight optical confinement of nanofibers; second, they have fast response times and small sample volumes owing to the high surface-to-volume ratio and low optical loss of nanofibers; third, they have high flexibility and stability due to the embedding of nanofibers in microfluidic chips or functional materials; and fourth, they have simple operation and low cost owing to the seamless connection between nanofibers and standard optical fibers. The disadvantages of nanofiber fluorescent sensors are: first, they have low selectivity and specificity owing to the broad spectrum and overlapping peaks of fluorescence signals; second, they experience interference from environmental factors such as temperature, humidity, and pH due to the lack of protective coating or shielding layers on the nanofibers; third, they have limited applications due to the difficulty of functionalizing nanofibers with different fluorescent reagents or probes.

Polymer nanofiber fluorescent sensors integrate new functional materials. They have many potential applications and challenges in various fields. However, they also require further improvement and exploration in several areas such as: their compatibility with biological systems, their anti-interference performance in complex environments, and their integration with other functional materials or devices. In addition, nanofiber fluorescent sensors have potential applications in many emerging fields, for example, using their luminescent properties under visible light or near-infrared light to achieve efficient detection or treatment of viruses or cancer cells. In addition, their response to electric or magnetic fields can be used to achieve the conversion or regulation of electromagnetic signals.

**Table 1:** Comparison of different types of nanofiber fluorescence sensors.

	Material	Application	Performance	Ref.
pH	poly(HEMA*-co-*NMA*-co-*RhBN2AM)	highly selective for pH and Hg^2+^	for Hg^2+^ is 10^−7^ M (LOD)	[86]
	BC	detect pH and glucose	for glucose is 0.026 mM (LOD)	[73]
	PNI	high sensitivity to pH (4–10)	pH (4–10) (detection range)	[74]
	PHEM-PNMA-PNBD	high sensitivity in sensing Fe^3+^ and pH	-	[88]
	PAN	for the pH of alkaline vapors and aqueous media	4.08 ppb (LOD)	[89]
Gas	PEO	tested as phosgene chemosensors	0.7–2.8 ppb (detection range)	[72]
	NFM	volatile organic compounds	3–40 μM (detection range)	[78]
	PMI	detection of various inert VOCs	1160 ppm to 116 ppm (detection range)	[75]
	TOCN, CD, TN	effectively detect and remove Cr (VI)	228.2 mg/g (maximum adsorption capacity)	[76]
	Phos-3	detection of phosgene in the gas phase	25 ppb (LOD)	[77]
	PMMA/PFO	monitor volatile organic compounds	47.9 and 15.4 ppm (LOD)	[90]
	PEO/MePyCz	fast response and high quenching efficiency towards DNT vapor	-	[91]
Biomolecules	PVA	detect prostate specific antigen (PSA)	0.46 pg·mL^−1^ (LOD)	[79]
	CA/Fe/CDs	detection of mercury (II) and lead (II) ions	-	[80]
	PVP	detect the nerve agent stimulant DECP	1.3 nM (LOD)	[81]
Explosives	Oligotriphenylene	detection of nitro-based explosives	1 nM (LOD)	[82]
	PVP/BaWO_4_	detection 2-nitrotoluene and H_2_O_2_	for 2-nitrotoluene is 1–400 ppb (detection range)	[83]
	PS/PyCz	detect nitro explosive vapors	91%, 90% and 94% (fluorescence quenching efficiencies)	[92]
Biogenic amines	TO-CNFs	detection limit for BAs was as low as 1 ppm	1 ppm (LOD)	[84]
	ethanol/chloroform	solid-state DNA based nanofibers can be an efficient matrix for FRET	-	[18]
Environment monitoring	NIPAAm	detection Hg^2+^, temperature, and magnetism	for Hg^2+^ is 10^−3^ M (LOD)	[93]
	Polyaniline	detection of As (III) in contaminated water	0.001 ppb (LOD)	[94]
	FNFM/PAN	the detection of mercuric ions (II)	1 ppb (LOD)	[95]
	CA/PCL	detection of Mercury ion	0.309 ppb (LOD)	[96]
	cellulose acetate	detecting Pb^2+^	0.16 ppb (LOD)	[97]
	PAN	sensors of Fe (III)	3.95 μM (LOD)	[98]
	Poly(NIPAAm- co -NMA- co -RHPMA)	sense Cu^2+^ both in solution and in solid state	1 × 10^−6^ to 1 × 10^−5^ M (detection range)	[99]

### 3.2. Raman Sensor

Raman sensors are sensors that use the Raman scattering phenomenon to detect target substances. Raman scattering is a nonelastic scattering phenomenon that occurs when incident light interacts with molecules and changes its frequency, which can reflect the vibration and rotation information of molecules. Raman sensors have the advantages of not requiring labeling, strong specificity, and the capacity to be used for detection in biology, chemistry, environmental monitoring, and other fields. According to the different enhancement mechanisms, nanofiber Raman sensors can be divided into surface-enhanced Raman spectroscopy (SERS) technology, tip-enhanced Raman spectroscopy (TERS) technology, shell-isolated nanoparticle-enhanced Raman spectroscopy (SHINERS) technology, and coherent anti-Stokes Raman spectroscopy (CARS) technology.

**Figure 11:**
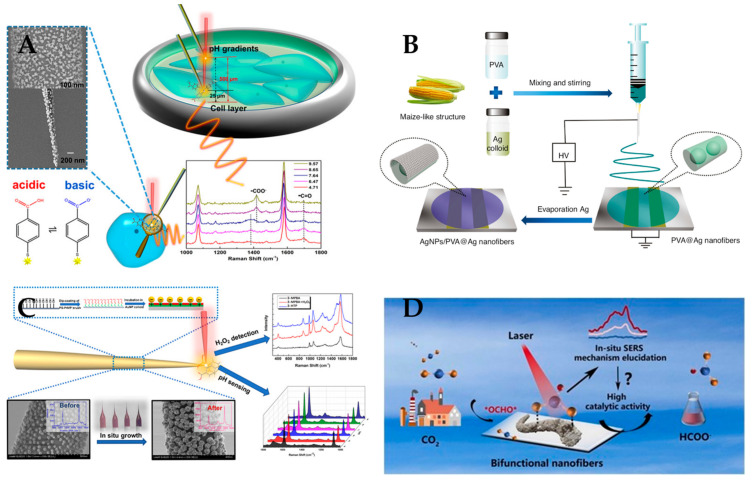
Different types of polymer nanofiber Raman sensors. (**A**) Raman sensors for pH detecting. Reproduced with permission from reference [15]. Copyright 2020, American Chemical Society. (**B**) Raman sensors for detecting the mixture of Sudan I, CV, and MG molecule. Reproduced with permission from reference [111]. Copyright 2019, De Gruyter. (**C**) Raman sensors for H_2_O_2_ detection and pH sensing. Reproduced with permission from reference [114]. Copyright 2020, American Chemical Society. (**D**) Raman sensors for corresponding to the key OCHO. Reproduced with permission from reference [115]. Copyright 2023, Elsevier.

The advantages of polymer nanofiber Raman sensors lead to their broad range of applications in different fields, as shown in Table 2. Figure 11 shows different types of polymer nanofiber Raman sensors for the detection of pH, the mixture of Sudan I, CV and MG molecules, and H_2_O_2,_ OCHO. (1) For biological applications, Zhao et al. [15], Zhu et al. [100], Turasa et al. [101], and Wang et al. [102] used nanofibers to detect various biological molecules, such as neurotransmitters, metabolites, toxins, and bacteria. They exploited the high surface area and biocompatibility of nanofibers to enhance the sensitivity and selectivity of their sensors. (2) For ion detection, Zhao et al. [103] and Zhang et al. [104] used nanofibers coated with metal nanoparticles to detect Hg^2+^ and Ag^+^ ions. They utilized the surface plasmon resonance and SERS effects of metal nanoparticles to amplify the signal of the target ions. (3) For dye detection, Chen et al. [105], Chen et al. [106], Juntaracena et al. [107], Nasr et al. [108], Sang et al. [109], Wan et al. [110], and Zhao et al. [111] used nanofibers decorated with metal nanoparticles or metal oxides to detect various dyes, such as Rhodamine 6G, Sudan I, Crystal Violet, Methylene Blue, etc. They employed the SERS effect of the nanofiber substrates to achieve high sensitivity and specificity for dye detection. (4) For gas detection, Qi et al. [112] used nanofibers to detect hydrogen gas. They designed a nanofiber sensor with ultrafast, ultrasensitive, and ultrawide dynamic range performance by using a palladium-coated silicon nanowire array embedded in a porous polycarbonate membrane. (5) For ion detection and pH detection, Chun et al. [113] used nanofibers to detect eccrine sweat pH, the wearable sensing platform based on electrospinning polyaniline nanofibers that can monitor the pH changes of sweat in real time. However, polymer nanofiber Raman sensors also have some shortcomings. First, they have low reproducibility and uniformity due to the random distribution and aggregation of nanofibers. Second, nanofibers may have structural defects or impurities that affect their Raman properties. Third, they have limited applications owing to the difficulty of functionalizing nanofibers with different Raman probes or labels.

Although Raman sensors have been widely used in many fields, polymer nanofiber Raman sensors face some challenges. For example, the integration of polymer nanofiber Raman sensors with other optical devices or systems, such as microfiber optics, fiber gratings, fiber lasers, and microfluidics, to achieve multifunctional and miniaturized sensing platforms, as well as the question of how to combine nanofiber Raman sensors with microfiber optics to enhance the light–matter interaction and improve the sensitivity and response time. Other challenges are how to integrate nanofiber Raman sensors with fiber gratings to realize wavelength-selective filtering and multiplexing of Raman signals, how to couple nanofiber Raman sensors with fiber lasers to achieve high-power and narrow-linewidth excitation sources for Raman sensing, and how to incorporate nanofiber Raman sensors into microfluidic systems to achieve rapid and precise manipulation and detection of liquid samples.

### 3.3. Surface Plasmon Resonance Sensor

Surface plasmon resonance (SPR) sensors use the excitation and propagation characteristics of surface plasmons at the metal–dielectric interface to detect target substances. Surface plasmons are electromagnetic waves formed by the resonance of free electrons on the metal surface with incident light. Polymer nanofiber optical sensors can achieve SPR sensing through modification of the surface or inside of nanofibers with metal nanoparticles or thin films, or by using nanofibers as excitation or detection elements of surface plasmons. SPR sensors have the advantages of high sensitivity, high resolution, and label-free detection. They can be used for detection in various fields, such as biology, chemistry, and environmental monitoring.

**Figure 12:**
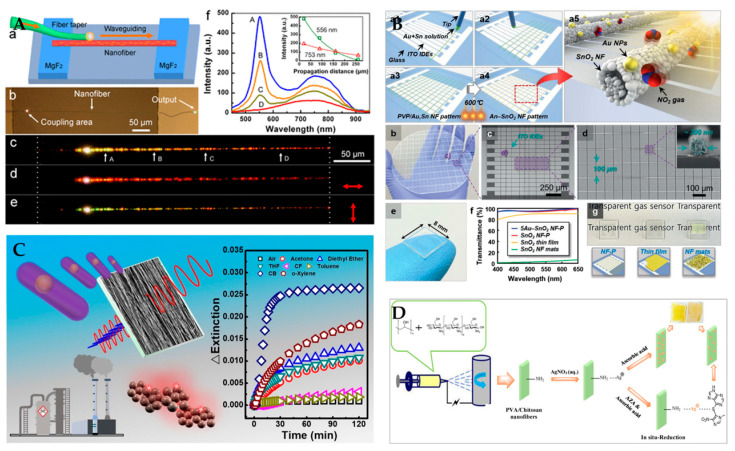
Different types of polymer nanofiber SPR sensors. (**A**) SPR sensors for humidity detecting. Reproduced with permission from reference [124]. Copyright 2012, American Chemical Society. (**B**) SPR sensors for NO_2_ detecting. Reproduced with permission from reference [119]. Copyright 2021, Wiley. (**C**) SPR sensors for volatile organic compounds detecting. Reproduced with permission from reference [120]. Copyright 2020, Elsevier. (**D**) SPR sensors for azathioprine determination. Reproduced with permission from reference [118]. Copyright 2018, Elsevier.

SPR sensors are used in many fields due to their unique advantages, as shown in Table 3. Figure 12 shows different types of polymer nanofiber SPR sensors for detecting humidity, NO2, organic compounds, and azathioprine. (1) For chemical detection, Rezaei et al. [118], Lim et al. [119], Wu et al. [120], and Marega et al. [121] used nanofibers to detect azithromycin, nitric oxide, volatile organic compounds, and biogenic amines. They exploited the high surface area and conductivity of nanofibers to achieve high sensitivity and selectivity for chemical detection. (2) For biomolecular detection: Nesuwan et al. [122] and Pule et al. [123] used nanofibers to detect human immunoglobulin G and oestrogenic compounds. They utilized the high affinity and biocompatibility of nanofibers to capture and identify the target biomolecules. (3) For environment monitoring, Wang et al. [124] used nanofibers embedded with gold nanorods to achieve optical waveguiding. They designed a nanofiber sensor with high flexibility and transparency for applications in optical communication and sensing. (4) For penitential applications, Bao et al. [125], Barakat et al. [126], Tsuboi et al. [127], and Shi et al. [128] exploited the SPR effect of metal nanoparticles to achieve tunable light scattering or absorption of the nanofiber sensors. They also demonstrated the applications of the polymer nanofiber sensors in chemical and biological sensing. However, SPR sensors have low reproducibility and uniformity due to the random distribution and aggregation of polymer nanofibers. They also have interference from background light or noise due to the weak SPR signals and low signal-to-noise ratio of nanofibers. In addition, it is difficult to control the polymer nanofiber diameter and orientation during fabrication, which can affect the SPR performance of nanofiber sensors.

Polymer nanofiber SPR sensors face several challenges related to the fabrication, characterization, and optimization of nanofibers and their integration with SPR sensing systems. The nanofiber fabrication techniques of nanofibers require precise control over the parameters that affect the nanofiber diameter, morphology, alignment, and uniformity. These parameters can influence the optical and mechanical properties of the polymer nanofibers and their interaction with the SPR signal. The characterization techniques of polymer nanofibers require high-resolution and high-contrast imaging methods to measure the nanofiber properties and quality. These methods can help to evaluate the polymer nanofiber structure, composition, distribution, and defects that can affect the SPR sensing performance. The optimization techniques of polymer nanofibers require careful selection of the materials and methods that can improve the polymer nanofiber performance and stability for specific sensing applications. These techniques can involve modifying the polymer nanofiber surface or core with metal nanoparticles or thin films, or using functional polymers or coatings to enhance the SPR signal or prevent degradation. Polymer nanofibers can introduce noise and interference to the SPR signal due to their scattering and absorption effects. Polymer nanofibers can also reduce the sensitivity of the SPR signal due to their low refractive index contrast with the surrounding medium. This can limit the dynamic range and resolution of SPR sensing.

### 3.4. Photoelectrochemical Sensor

Photoelectrochemical sensors use photosensitive substances doped or coated in nanofibers to produce current or voltage signals when irradiated with light. These signals can be used to perform electrochemical analysis of target substances. Polymer nanofiber photoelectrochemical sensors can provide a large number of active sites and channels, which can increase the contact opportunity and reaction rate with the measured substance. This can improve the sensitivity and response speed of the photoelectrochemical sensors.

**Figure 13:**
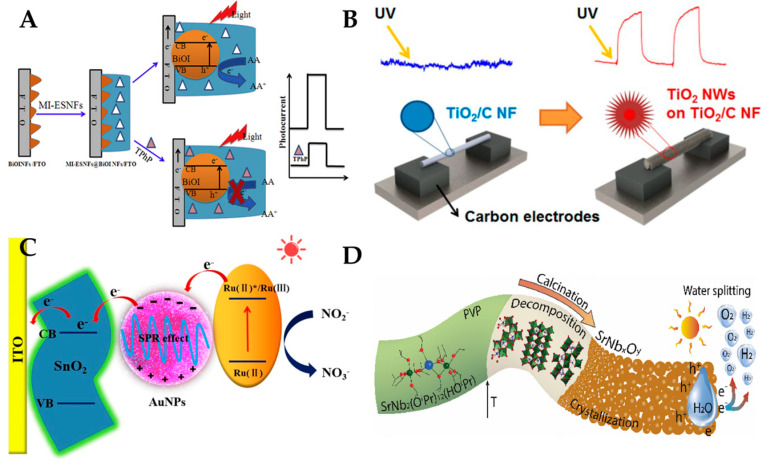
Different types of polymer nanofiber photoelectrochemical sensors. (**A**) Photoelectrochemical sensors for detection of triphenyl phosphate. Reproduced with permission from reference [129]. Copyright 2017, Elsevier. (**B**) Photoelectrochemical sensors for use with UV and pH. Reproduced with permission from reference [133]. Copyright 2014, American Chemical Society. (**C**) Photoelectrochemical sensors for determination of NO_2_^−^. Reproduced with permission from reference [130]. Copyright 2020, Elsevier. (**D**) Photoelectrochemical sensors for water splitting. Reproduced with permission from reference [139] Copyright 2020, Elsevier.

Owing to their unique advantages, photoelectrochemical sensors are used in many fields, as shown in Table 4. Figure 13 shows different types of polymer nanofiber photoelectrochemical sensors for the detection of triphenyl phosphate, UV, pH, NO^2−^, and water splitting. (1) For environmental monitoring, Yang et al. [129] Luo et al. [130], Kokulnathan et al. [131], and Cui et al. [132] used electrospinning nanofibers to detect various environmental pollutants, such as triphenyl phosphate, metal ions, volatile organic compounds, and silver tungstate. They exploited the photoelectrochemical, electrochemical, or optical properties of nanofibers to measure the concentration or presence of the pollutants in water or air samples. (2) For biomedical analysis: Zhang et al. [17], Lee et al. [133], Shaibani et al. [134], Farzin et al. [135], Shaibani et al. [136], and Xie et al. [137] used electrospinning nanofibers to detect various biomedical analytes, such as hydrogen peroxide, bacteria, pathogen outbreaks, dyes, cancer cells, and mercury ion. They utilized the photoelectrochemical, electrochemical, or optical properties of nanofibers to measure the level or activity of the analytes in biological samples. (3) For energy conversion: Mei et al. [138] and Graf et al. [139] used electrospinning nanofibers to convert chemical or solar energy into electrical energy. They employed the electrochemical or photoelectrochemical properties of nanofibers to generate electric currents or voltages from chemical reactions or light irradiation. However, polymer nanofiber photoelectrochemical sensors also have some shortcomings. First, nanofiber photoelectrochemical sensors are prone to interference from other substances in the environment. Second, nanofiber photoelectrochemical sensors can suffer from photodegradation or photocorrosion of the photoactive materials, which may affect their stability and reproducibility. Third, the morphology of polymer nanofibers on the surface of the sensor directly affects the performance, so a more complex polymer nanofiber integration process is required to improve sensor performance.

The challenges facing polymer nanofiber photoelectrochemical sensor research are overcoming the mass transfer limitation of triphase (gas–liquid–solid) reactions, achieving stable and durable operation under harsh conditions, and integration with sensing devices and detection modes for practical applications.

### 3.5. Other Types of One-Dimensional Nanofiber Optical Sensors

In addition to the types outlined above, there are other types of polymer nanofiber optical sensors, such as interferometric, reflective, refractive, and absorptive sensors. These types of optical sensors use nanofibers to produce interference, reflection, refraction or absorption effects on incident light to achieve sensing. These effects are related to the structure, morphology, composition, arrangement, and other factors of the nanofibers, so they can be used to detect the material changes around or inside the nanofibers. These types of polymer nanofiber optical sensors have the advantages of simple structure, easy preparation, low cost, etc. They can be used to detect physical or chemical quantities such as temperature, humidity, pressure, strain, and pH.

**Figure 14:**
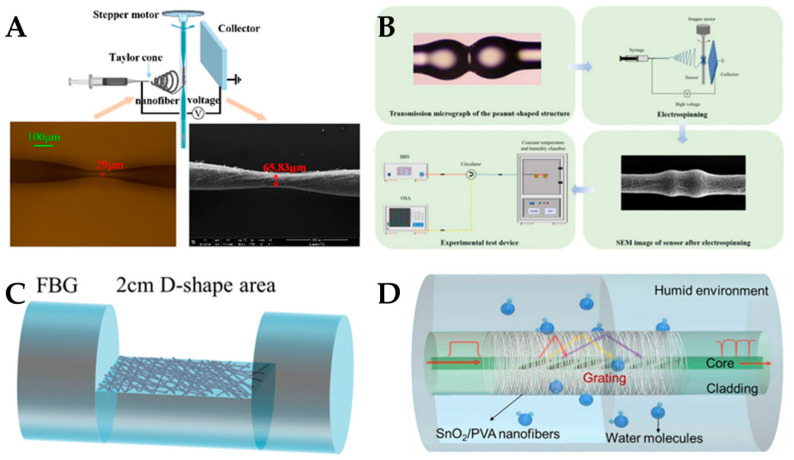
Different types of fiber optic sensors with integrated nanofibers. (**A**) Nanofiber optical fiber sensor for humidity sensing. Reproduced with permission from reference [140]. Copyright 2020, MDPI. (**B**) Nanofiber optical fiber sensor for temperature sensing. Reproduced with permission from reference [141]. Copyright 2022, Elsevier. (**C**) Nanofiber optical fiber sensor for glucose sensing. Reproduced with permission from reference [142]. Copyright 2021, IEEE. (**D**) Nanofiber optical fiber sensor for breath monitoring. Reproduced with permission from reference [143]. Copyright 2023, Elsevier.

In recent years, with the continuous development of optical fiber sensing technology, the combination of micro-nano structured optical fibers and polymer nanofibers has led to additional unique sensing characteristics. Our findings in this area are shown in Figure 14. In terms of environmental monitoring, the detection of humidity is achieved by combining PVA nanofibers with carbon nanotubes, and fixing them on the surface of micro-nano tapered optical fibers [140]. We also realized simultaneous detection of ambient temperature and humidity by integrating PVA/nano-ZnO nanofibers on the surface of the micro-nano peanut-shaped structure. The experimental results show that the surface-integrated nanofiber sensor has a higher response speed [141]. In terms of biosensing detection, glucose oxidase is encapsulated in PVA nanofibers and integrated on the surface of D-shaped optical fiber to realize the detection of glucose [142]. At the same time, we also realize the detection of patients’ breath by integrating PVA/nano-SnO2 on the surface of TFBG [143].

In summary, polymer nanofibers have significant potential for application in optical fiber sensing owing to their unique structures and properties. We believe that the special optical properties of nanofibers, coupled with the unique micro-nano structure of optical fibers, will be broadly applied in the preparation of various types of ultrasensitive micro-nano structure sensors in the future. These sensors are also an important platform for the development of nanofiber microstructure sensors in the future.

## 4. Applications and Prospects of Nanofiber Optical Sensors

The nanofiber sensors can achieve highly sensitive and selective detection in the fields of biomedicine, environmental monitoring, food safety, and energy conversion, which will lead to a more convenient and generally improved human experience.

### 4.1. Biomedicine

In the field of biomedicine, polymer nanofiber optical sensors can be used for the detection and diagnosis of biomolecules, cells, tissues, and diseases. The nanofiber sensors can be used in protein detection, biocatalysts, biological test strip sensing platforms, microRNA detection, bacteria detection, etc. In the future, through more research on the functionalization and modification of nanofibers, more types of nanofiber biomedicine sensors with excellent performance are expected to be obtained.

**Figure 15:**
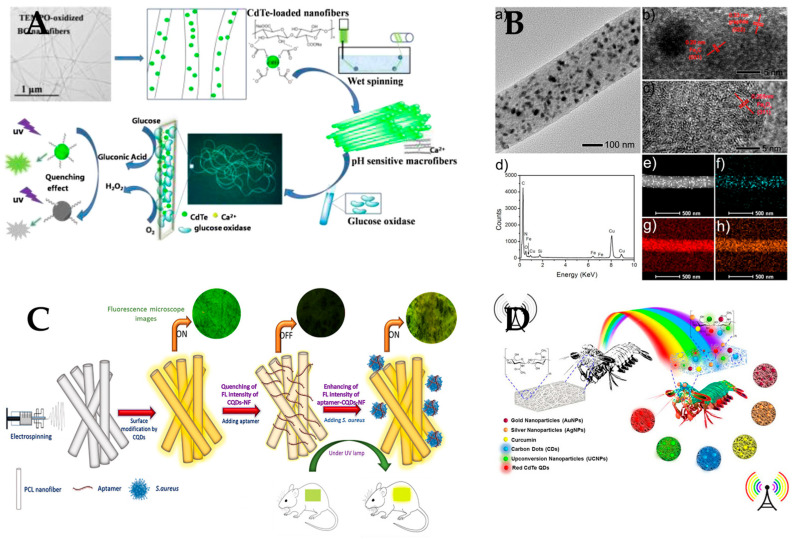
Application of polymer nanofiber optical sensor in the biomedicine field. (**A**) Nanofiber sensors for pH and glucose sensing. Reproduced with permission from reference [73]. Copyright 2018, Elsevier. (**B**) Nanofiber sensors as highly efficient biocatalyst with oxidase-mimicking activity. Reproduced with permission from reference [147]. Copyright 2018, American Chemical Society. (**C**) Nanofiber sensors for detection of pathogenic bacteria in the wound. Reproduced with permission from reference [151]. Copyright 2022, Elsevier. (**D**) Chitin nanofiber paper for optical biosensing applications. Reproduced with permission from reference [148]. Copyright 2020, American Chemical Society.

Figure 15 shows the application of polymer nanofiber optical sensor in biomedicine field for the detecting of the pH, glucose, biocatalyst, pathogenic bacteria, and optical biosensing. (1) For protein detection, Davis et al. [144] reported a sensor that uses an anionic fluorescent dendrimer (AFD) encapsulated in cellulose nanofibers to detect proteins by fluorescence quenching. Lee et al. [145] reported the use of an aptamer-immobilized electrospinning polystyrene–poly (styrene-co-maleic anhydride) (PS–PSMA) nanofiber as a new aptasensor platform for protein detection. Kirbay et al. [146] synthesized and characterized L1AN-FeMOF as a probe for anti-CRP labelling and fabricated PCL/PAA nanofibers for their application in immunoassay preparation. (2) For detection of glucose in blood, Yao et al. [73] focused on the fabrication of color-tunable luminescent macrofibers based on CdTe-loaded bacterial cellulose (BC) nanofibers by wet spinning, and their optical properties. (3) For biocatalysts, Song et al. [147] reported the synthesis of Fe3C nanoparticles encapsulated within nitrogen-doped carbon (Fe3C/N–C) nanofibers as efficient biocatalysts for sensing applications. (4) For a biological test strip sensing platform, Naghdi et al. [148] exploited the beneficial properties of chitin nanofiber (ChNF) paper to fabricate transparent, efficient, biocompatible, flexible, and miniaturized optical sensing bioplatforms via embedding/immobilizing various plasmonic nanoparticles and colorimetric reagents in the 3D nanonetwork scaffold of the ChNF paper. (5) For microRNA detection, Chavoshy et al. [149] developed an optical platform based on fluorescent polyacrylonitrile nanofiber for the detection of microRNA-21 as a biomarker of cancerous cells. Fu et al. [150] reported a novel FRET miRNA-195-targeting biosensor, based on silica nanofibers incorporated with rare earth-doped calcium fluoride particles (CaF2:Yb, Ho@SiO2) and gold nanoparticles (AuNPs), that triggers the FRET effect when the target miRNA is captured by oligonucleotides conjugated at the surface of CaF2:Yb,Ho@SiO2 fibers and AuNPs. (6) For bacteria detection, Pebdeni et al. [151] introduced a novel and smart nanofiber network with the electrospinning method to create an amplified fluorescent biosensing platform for the detection of Staphylococcus aureus (*S. aureus*) bacteria in wounds. Ghasemi et al. [152] evaluated the development of a fluorescent electrochemical biosensor for the identification of Streptococcus agalactiae. Zhang et al. [153] demonstrated a novel approach for fabricating SERS substrates for single bacterial biosensing based on Ag cylindrical nanotrough networks (CNNs) using a cellulose nanofiber template fabrication via facile electrospinning.

However, polymer nanofiber optical sensors for biomedicine also face some challenges, such as: (1) the fabrication of nanofibers with uniform size, shape, and composition; (2) the integration of nanofibers with other components, such as electrodes, transducers, or microfluidic channels; (3) the stability and durability of nanofibers under different environmental conditions, such as temperature, humidity, or pH; and (4) the biocompatibility and toxicity of nanofibers and their functionalization agents. We therefore hope that research in the field of biomedicine sensing will focus on the unique advantages and potential of nanofibers in sensing. Addressing the current difficulties will lead to more types of high-performance sensors for future applications in various fields of biomedicine.

### 4.2. Environmental Monitoring

In the field of environmental monitoring, polymer nanofiber optical sensors can be used for the detection and evaluation of air quality, water quality, soil quality, etc. The nanofiber sensors can be used in the fields of water pollution detection, gas sensing, organic/inorganic pollutant detection, magnetic field, and temperature sensing. These sensors have high specific surface area and porosity, which can enhance the contact and interaction with environmental target analytes, and improve detection sensitivity and response speed.

**Figure 16:**
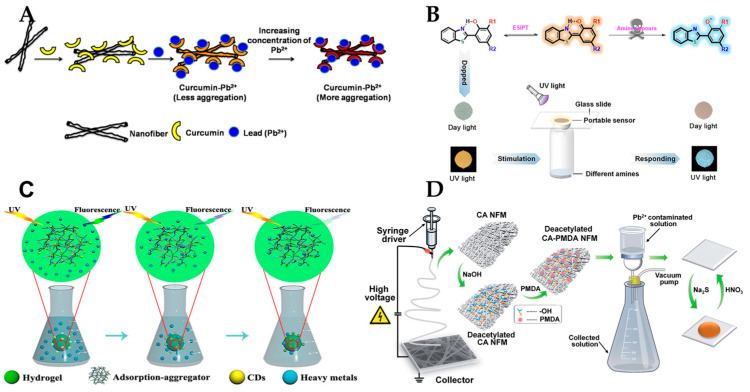
Application of polymer nanofiber optical sensors in the environmental monitoring field. (**A**) Nanofiber sensors for lead ion detection. Reproduced with permission from reference [155]. Copyright 2016, Elsevier. (**B**) Nanofiber sensors for detection of amine/ammonia. Reproduced with permission from reference [159]. Copyright 2021, Elsevier. (**C**) Nanofiber sensors for highly efficient adsorption and detection of Cr (VI). Reproduced with permission from reference [162]. Copyright 2021, Elsevier. (**D**) Nanofiber sensors for detection and removal of lead (II) ions. Reproduced with permission from reference [156]. Copyright 2015, Royal Society of Chemistry.

Figure 16 shows the application of polymer nanofiber optical sensors in the environmental monitoring field for the detection of ions, amine, Cr (VI), and lead (II) ions. (1) For water pollution detection, Chen et al. [154] describes a promising sunlight-driven photocatalyst for the treatment of ofloxacin and other fluoroquinolone antibiotics in water and wastewater. Raj et al. [155] developed a simple, biocompatible, and selective colorimetric sensor strip for detection of lead (Pb^2+^) using curcumin loaded cellulose acetate (CC-CA) nanofibers. Li et al. [156] fabricated a solid-phase nanofibrous material for Pb^2+^ detection using pyromellitic dianhydride (PMDA) modified deacetylated cellulose acetate membranes (DCA-PMDA). (2) For gas sensing, Wen et al. [157] proposed a humidity sensor based on graphene quantum dot nanofibers on a U-shaped optical fiber. Cai et al. [158] introduced a concept of utilizing functional film-coated optical micro-/nanofibers for gas sensing, and demonstrated a humidity sensor using crystal violet (CV)-doped Nafion film. Zheng et al. [159] synthesized a nanofiber-based sensor for volatile amine vapors using polyacrylonitrile (PAN) and polyvinyl alcohol (PVA) as matrix materials, and 4-aminothiophenol (4-ATP) as the sensing agent. (3) For ecological environment monitoring, Xue et al. [160] created nanofibers consisting of either a poly (ether sulfone) (PES) or a polysulfone (PSU) core coated by a biocompatible polycaprolactone (PCL) shell, and incorporated oxygen-sensitive luminescent probes Pt(II) meso-tetra (pentafluorophenyl) porphine (PtTFPP) or Pd(II) meso-tetra (pentafluorophenyl) porphine (PdTFPP) in the core via single-step coaxial electrospinning. Shehata et al. [161] prepared a nanocomposite of cerium oxide (ceria) nanoparticles embedded in electrospinning PVA nanofibers for optical sensing of radicals in solutions. Yuan et al. [162] synthesized a fluorescent lignin-based hydrogel with cellulose nanofibers and carbon dots (CDs) for the control of hexavalent chromium (Cr(VI)). Jin et al. [163] designed a rhodol-based ratiometric fluorescent probe for the reversible recognition of Cu^2+^ and ATP under theoretical analysis. (4) For organic/inorganic pollutant detection, Lokesh et al. [164] investigated the role of a nanostructured n-ZnO/p-NiO heterostructure as a room temperature ammonia sensor. Wei et al. [165] developed a new terbium(III) organic framework for the effective detection of antibiotics, nitro-compounds, excessive Fe^3+^, and MnO_4_. Teera et al. [90] analyzed the changes in luminescence properties of PMMA_PFO nanofibers when exposed to volatile organic compounds (VOCs). (5) For magnetic field and temperature sensing, Li et al. [166] proposed a novel sensor for the simultaneous detection of magnetic fields and temperature by using a micro-nanofiber Mach Zehnder interferometer (MZI). The development of nanofiber-based sensors is crucial, and plays an important role in a wide range of applications, especially in environmental monitoring. With the further development of various fabrication techniques, nanofiber sensors with excellent filtering, photocatalytic, and sensing properties have been produced, which can offer important application prospects for environmental monitoring.

However, other challenges faced by polymer nanofiber optical sensors in the field of environmental monitoring are: (1) the calibration and validation of nanofiber optical sensors for different environmental conditions and analytes; (2) the interference and cross-sensitivity of nanofiber optical sensors to multiple environmental factors, such as humidity, temperature, pressure, and light intensity; (3) the fabrication of nanofiber optical sensors with high reproducibility and scalability; and (4) the standardization and regulation of nanofiber optical sensors for environmental monitoring applications. These challenges require further research and innovation to enhance the reliability and applicability of nanofiber optical sensors in environmental monitoring.

### 4.3. Food Safety

In the field of food safety, polymer nanofiber optical sensors can be used for the detection and analysis of harmful substances, microorganisms, nutrients, etc., in food. The polymer nanofiber sensors can be used in the fields of food freshness detection, water pollution detection, gas sensing, pollutant residue detection, etc. We believe that in the future, the advantages of low cost and high manufacturing efficiency of nanofibers can be used to prepare more types of sensors for food safety detection, and to protect human diet health.

**Figure 17:**
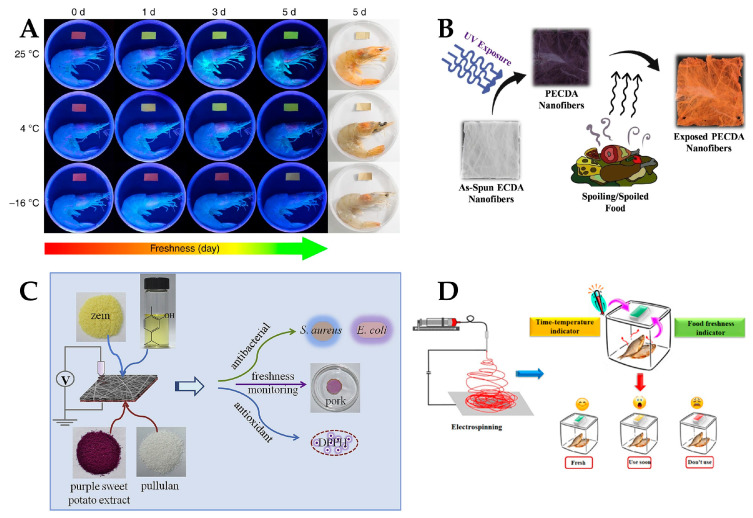
Application of polymer nanofiber optical sensors in the food safety field. (**A**) Nanofiber sensors for detection of shrimp and crab freshness. Reproduced with permission from reference [167]. Copyright 2019, Springer. (**B**) Nanofiber sensor for food spoilage detection. Reproduced with permission from reference [170]. Copyright 2019, Elsevier. (**C**) Nanofiber sensors for food freshness monitoring and preservation. Reproduced with permission from reference [168]. Copyright 2019, Elsevier. (**D**) Nanofiber sensors in food intelligent packaging. Reproduced with permission from reference [182]. Copyright 2021, Royal Society of Elsevier.

Figure 17 shows the application of polymer nanofiber optical sensors in the food safety field for the detection of the crab freshness, food spoilage, food freshness, and food intelligent packaging. (1) For food freshness detection, Jia et al. [167] designed and prepared cellulose-based ratiometric fluorescent materials with a superior amine-response, which offers the real-time and visual detection of seafood freshness. Guo et al. [168] developed novel intelligent double-layer fiber mats via the electrospinning technique for pork freshness monitoring and preservation, using pullulan-purple sweet potato extract (PL-PSPE) and zein-glycerol-carvacrol (ZN-GL-CA) as the functional layers. Aghaei et al. [169] described a halochromic sensor of cellulose acetate nanofibers and alizarin as a fish spoilage indicator in real-time, which changes color with an increase in the amount of total volatile basic nitrogen (TVB-N) and a rise in the pH value of product. Valdez et al. [170] demonstrated optical sensing of biogenic amines (BAs) using silica-reinforced polydiacetylene (PDA) nanofiber mats by a novel force-spinning technique. Quan et al. [84] designed a nanofiber ratiometric fluorescent sensor for BAs using cellulose nanofibers (CNFs) as the skeleton, and fluorescein isothiocyanate and proporphyrin IX modified nanofibers as the indicator and internal reference, respectively. Yildiz et al. [171] developed an electrospinning nanofiber halochromic pH sensor film using curcumin, chitosan (CS), and polyethylene oxide (PEO) to monitor chicken freshness. Kiryukhin et al. [172] developed a membrane film sensor (MFS) to measure the pH of fluids, which comprises a polyelectrolyte multilayer film with uniformly distributed compartments (microchambers) where a fluorescent sensing dye is encapsulated. (2) For water pollution detection, He et al. [173] reported a simple nanofiber-based platform for highly sensitive colorimetric/fluorometric detection of Escherichia coli (E. coli) using nanofiber membranes (NFM) loaded with target molecules (fluorescent and chromogenic substrate) via chemical modification. Nag et al. [174] developed an optical enzymatic biosensor for rapid and point-of-use detection of β-lactam antibiotics in water by immobilizing horseradish peroxidase onto electroactive polyaniline nanofibers. Zhang et al. [175] presented a simple nanofiber-based platform for highly sensitive colorimetric/fluorometric detection of Escherichia coli (E. coli) using nanofiber membranes (NFM) loaded with target molecules (fluorescent and chromogenic substrate) via chemical modification. Shaibani et al. [176] reported a light addressable potentiometric sensor integrated with electrospinning poly acrylic acid/polyvinyl alcohol (PAA/PVA) hydrogel nanofibers as a sensing layer (NF-LAPS) for detection of E. coli in orange juice. Abedalwafa et al. [177] prepared portable colorimetric biosensor strips by combining aptamer-immobilized electrospinning nanofiber membranes (A-NFMs) with signal probes (DNA-conjugated gold nanoparticles (AuNPs)) for determination of kanamycin (KMC) as a model analyte. (3) For gas sensing, Pirsa et al. [178] prepared an ethylene optical sensor using bacterial cellulose (BC) loaded with potassium permanganate (KMnO4), which was used for detection and determination of ethylene concentration in bunch banana packages. (4) For pollutant residue detection, Teixeira et al. [179] developed a droplet-based optofluidic system for the detection of foodborne pathogens, combining the loop-mediated isothermal amplification (LAMP) technique with surface-enhanced Raman scattering (SERS). Nguyen et al. [180] reported a method for detecting alcohol concentration using a PVA nanofiber scaffold as the immobilized sensing film in samples of wine, beer, or some alcoholic beverages. Luo et al. [181] reported a chemiluminescence biosensor for hydrogen peroxide determination by immobilizing horseradish peroxidase onto a PVA-co-PE nanofiber membrane. The outlined studies show that nanofiber sensors have been used in the food industry for rapid food detection. Food safety is an important issue that affects everyone. Therefore, it is very important to develop various types of nanofiber sensors that can quickly determine food safety.

However, polymer nanofiber optical sensors for food safety also face some challenges, including: (1) the functionalization and immobilization of nanofibers with specific biorecognition elements such as antibodies, enzymes, and DNA probes; (2) the stability and reproducibility of nanofiber optical sensors in complex food matrices and environmental conditions; (3) the standardization and validation of nanofiber optical sensors for food safety analysis; (4) the selectivity and specificity of nanofiber optical sensors for different food contaminants in the presence of interfering substances; (5) the scalability and cost-effectiveness of nanofiber optical sensors for large-scale production and commercialization. Therefore, more research and development are needed to overcome these challenges, and to optimize the performance of nanofiber optical sensors for food safety applications.

### 4.4. Development and Prospect

Nanofiber optical sensors are a promising technology that can offer high sensitivity, fast response, and compact size for various sensing applications, and have the following advantages. (1) High specific surface area and porosity: polymer nanofibers can provide high specific surface area and porosity, thereby enhancing the contact and interaction between the sensor and the target substance, and improving the sensitivity and response speed of the sensor. (2) Adjustable optical properties: polymer nanofiber materials can achieve adjustable optical signals through control of their composition, morphology, size, arrangement, and other parameters, thereby meeting the design requirements of different types of sensors. (3) Good mechanical properties and stability: polymer nanofibers can provide good mechanical properties and stability, thereby enhancing the durability and reliability of the sensor, and reducing the failure rate and maintenance costs. (4) Biocompatibility and environmental friendliness: polymer nanofibers can be prepared using renewable biomass materials, thereby providing biocompatibility and environmental friendliness, reducing the harm of the sensor to the human body and environment, and conforming to the concept of green and sustainable development.

However, polymer nanofiber optical sensors also face the following limitations. (1) Fabrication methods: one of the challenges in nanofiber optical sensor development is fabricating them with high quality, low cost, and scalability. There are different methods for producing nanofibers, including drawing, tapering, electrospinning, and chemical vapor deposition. Each method has its own advantages and disadvantages in terms of speed, precision, uniformity, and compatibility with different materials. Therefore, it is important to develop new fabrication techniques and improve existing ones to enhance the performance and functionality of nanofiber optical sensors. (2) Integration and packaging: another challenge is integrating nanofiber optical sensors with other components and devices, such as light sources, detectors, microfluidics, and electronics. Nanofibers are fragile and sensitive to environmental factors such as temperature, humidity, and mechanical stress. They therefore need to be protected and stabilized by proper packaging and encapsulation. Moreover, they need to be efficiently coupled with other optical elements, such as lenses, gratings, and waveguides. Therefore, it is important to design and optimize the integration and packaging of nanofiber optical sensors to enhance their robustness and reliability. (3) Functionalization and modification: one area of nanofiber optical sensor research focuses on functionalizing and modifying them with different materials and structures to achieve specific sensing functions and properties. Nanofibers have large surface-to-volume ratios and accessible evanescent fields, which make them ideal for attaching various functional molecules, nanoparticles, nanowires, nanofilms, or 2-D materials. These functional materials can introduce new optical effects and interactions, such as SPR, fluorescence, Raman scattering, and nonlinear optics. Therefore, it is important to explore different functionalization and modification methods to expand the sensing capabilities and applications of nanofiber optical sensors.

In the future, nanofiber optical sensors require further research and improvement in the following areas. (1) Optimization and innovation of the preparation process: simpler, fast, low-cost, and environmentally friendly preparation processes must be established to improve the quality and consistency of nanofiber materials, and achieve multifunctional integration and modularization of sensors. (2) Nanofiber optical sensors have wide-ranging development prospects and application potential. They can achieve high sensitivity selective detection in biology, chemistry, environmental monitoring, and other fields. Through continued research and development, polymer nanofiber optical sensors are expected to demonstrate high performance over a wide range of applications.

## 5. Conclusions

In order to further improve the performance and application range of polymer nanofiber optical sensors, future research with the following focuses should be undertaken: (1) Develop new types of nanofiber materials, such as nanofibers with novel structures, or functions, or composite, or heterogeneous structures of different types of nanofibers, to achieve multimode or synergistic modulation of light, thereby improving the sensitivity and selectivity of the sensors. (2) Optimize the preparation and detection methods of nanofiber optical sensors, such as using more accurate and controllable electrospinning or other methods to prepare nanofibers, or using more advanced and convenient light sources, photodetectors or spectrometers to detect the sensing signal, to improve the stability and repeatability of the sensors. (3) Explore the feasibility and adaptability of nanofiber optical sensors in practical applications, such as considering the interference factors or complex environment that may exist in actual samples on the sensing signal, or considering the remote control, or wireless communication functions that may be needed in actual detection, to improve the signal-to-noise ratio and intelligence level of the sensors. (4) Expand the application potential of nanofiber optical sensors in new fields or scenarios, such as using nanofiber optical sensors to detect new or difficult-to-detect target substances (such as COVID-19 virus). Using nanofiber optical sensors to achieve new or difficult-to-achieve functions (such as wearable or implantable). To achieve multimode or synergistic modulation of light, thereby improving the sensitivity and selectivity of the sensors.

In conclusion, polymer nanofiber optical sensors are a new type of optical sensor that have a broad application potential and value in biomedicine, environmental monitoring, food safety, and other fields. However, polymer nanofiber optical sensors also face challenges, for example, the complexity and cost of the preparation process, difficult signal conversion and readout, and signal interference and suppression, which require further research and improvement. In the future, polymer nanofiber optical sensors need to be further developed through preparation process optimization and innovation, simplification and improvement of signal conversion and readout, elimination of signal interference and suppression, and function expansion and integration. We believe that higher performance and wider application of polymer nanofiber optical sensors will enhance convenience and well-being throughout society.

## Figures and Tables

**Table 2:** Comparison of different types of nanofiber Raman sensors.

	Material	Application	Performance	Ref.
Biology	AuNRB/AuNS(S)	capturing cell pH heterogeneity	pH 6.5–9.5 (detection range)	[15]
	Zein	zein nanofiber-based flexible SERS platform	2.06 ng/mL (LOD)	[101]
	Ag-TEA@SiO_2_	detection of biomacromolecules of bacteria	10^−10^ mol/L (LOD)	[102]
	PVA-SbQ	(SERS)-based immunoassay	-	[116]
Ion	4-Mpy/PS-P4VP	detect Hg^2+^ and Ag^+^	5 nM–5 μM (detection range)	[103]
	gluten/zein film	to assay five common nitrite foods	1 ppm (LOD)	[104]
	PVA/Ag	Rhodamine 6 G detecting	1.3 nM (LOD)	[117]
Dye	AgNPs/PVA@Ag	detection of Crystal Violet	-	[111]
	PVDF	SERS sensing of molecules and bacteria	1160 ppm to 116 ppm (detection range)	[105]
	PVA/PEI	detection of enrofloxacin	-	[106]
	Ag/WO_3_/PVA	SERS substrates with recyclability	0.001 ppb (LOD)	[107]
	CoTiO_3_@Ag/PVP	detect R6G	10^−9^–10^−3^ M (detection range)	[108]
	PMMA/P4VP	using 4-MBA as probe molecule	10^−3^ M (LOD)	[109]
	Ag@TA@SiO_2_	identify *S. aureus*	0.46 pg·mL^−1^ (LOD)	[110]
Gas	SiO_2_	hydrogen detection	3 ppm (LOD)	[112]
pH	Au/TPU	SERS pH sensor	for Hg^2+^ is 10^−7^ M (LOD)	[113]

**Table 3:** Comparison of different types of nanofiber SPR sensors.

	Material	Application	Performance	Ref.
Chemical	Au-SnO_2_	detect ppb-level NO_2_	6 ppb (LOD)	[119]
	PMMA	detect VOCs below lowest explosion limits	100 ppm (LOD)	[120]
	PVA/CS	determination human serum samples	0.09 μM (LOD)	[118]
	PVA	detect biogenic amines from food vapors	10 ppm (LOD)	[121]
Biomolecule	PAA	the detection of human immunoglobulin G	15000 RU (sensitivity)	[122]
	polystyrene	the detection of oestrogenic compounds	100 ng/mL (sensitivity)	[123]
Environment monitoring	PAM/GNR	detect humidity	110 ms (response time)	[124]

**Table 4:** Comparison of different types of polymer nanofiber photoelectrochemical sensors.

	Material	Application	Performance	Ref.
Environmental monitoring	chitosan	detection of triphenyl phosphate	0.008 ppb (LOD)	[129]
	PVP	detect nitrite (NO_2_^−^) under visible light irradiation	4.8 × 10^−10^ M (LOD)	[130]
	PVP/Ag_2_WO_4_/WO_3_	Ag_2_WO_4_/WO_3_ photocatalyst was synthesized through electrospinning process combined with a facile solid chemical reaction.	-	[132]
Biomedical	WO_3_@TiC/C	detection of hydrogen peroxide	386 μA mM^−1^ cm^−2^ (sensitivity)	[17]
	PVP/TTIP	utilized as UV and pH sensors	5.68 ± 0.28 nS/pH (sensitivity)	[133]
	PVA/PAA	detect Escherichia coli	10^2^ CFU/mL (LOD)	[134]
	PVA/PAA	measures cancer cell metabolism and their response to anticancer drugs	74 mV/pH (sensitivity)	[136]
Energy conversion	PVP/SrNb_2_O_6_	oxide nanofiber meshes as potential photoanode material for solar water splitting	-	[139]

## Abbreviations

PEO	Poly (ethylene oxide)
TO-CNFs	TEMPO- oxidized cellulose nanofibers
PMI	Poly(α-methyleneindane)
CD	Carbon dot
RhBN2AM	Rhodamine derivative
PVA	Poly(vinyl alcohol)
CA	Cellulose acetate
PMMA_PFO	Poly(methyl methacrylate)_polyfluorene
NIPAAm	N-isopropylacrylamide
PVP	Polyvinyl pyrrolidone
BaWO_4_	Barium tungsten oxide
TN	Titanate nanofibers
PAN	Polyacrylonitrile
PS/PyCz	PS/PyCz polystyrene/9-(pyren-1-yl)-9H-carbazole
BC	Bacterial cellulose
FNFM	Fluorescent nanofibrous membrane
NFM	Nanofibrous membrane
PNI	Poly-1,8-naphthimide
PCL	Polycaprolactone
MePyCz	4-(2-(2-(2-methoxyethoxy)ethoxy)ethoxy)-9-(pyren-1-yl)-9H-carbazole
Poly(NIPAAm- co -NMA- co -RHPMA)	Poly[(N-isopropylacrylamide)-co-(N-hydroxymethyl acrylamide)-co-(4-rhodamine hydrazonomethyl-3-hydroxy-phenyl methacrylate)]
PHEM-PNMA-PNBD	Poly(2-hydroxyethyl methacrylate-co-N-methylolacrylamide-co-nitrobenzoxadiazolyl derivative)
4-Mpy	4-mercaptopyridine
PS-P4VP	Polystyrene-b-poly(4-vinyl pyridine)
PVDF	Poly(vinylidene fluoride)
TPU	Thermoplastic polyurethane
TEA	Triethanolamine
SbQ	N-methyl-4(4′-formylstyryl) pyridinium methosulfate acetal
CS	Chitosan
PAA	Poly(acrylic acid)
Poly(NIPAAm- co -NMA- co -RHPMA)	Poly[(N-isopropylacrylamide)-*co*-(*N*-hydroxymethyl acrylamide)-*co*-(4-rhodamine hydrazonomethyl-3-hydroxy-phenyl methacrylate)]
